# Nonattenuated Polymyxin B Used for the Treatment of Extreme-Drug Resistant *Acinetobacter baumannii*-Related Infections in Patients with Preexisting End Stage Renal Failure

**DOI:** 10.1155/2014/573279

**Published:** 2014-06-03

**Authors:** Yvonne Peijun Zhou, Nathalie Grace Sy Chua, Maciej Piotr Chlebicki, Winnie Hui Ling Lee, Andrea Lay Hoon Kwa

**Affiliations:** ^1^Department of Pharmacy, Singapore General Hospital, Outram Road, Singapore 169608; ^2^Department of Infectious Diseases, Singapore General Hospital, Outram Road, Singapore 169608; ^3^Duke-NUS Emerging Infectious Diseases, 8 College Road, Singapore 169857

## Abstract

Recent pharmacokinetic studies have suggested that nonrenal clearance predominates the elimination of polymyxin B. We present 2 patients with preexisting end stage renal failure, who were given nonattenuated doses of polymyxin B for the treatment of extreme-drug resistant organism. No evidence of adverse events occurred and microbiological clearance was documented.

## 1. Introduction 

Polymyxin B is a polypeptide antibiotic that was made commercially available in the late 1950s but its use fell out of favor over concerns of adverse events including nephrotoxicity and neurotoxicity [1]. However, the increasing prevalence of extensively-drug resistant (XDR) gram-negative bacteria, including* Acinetobacter baumannii, Pseudomonas aeruginosa, Klebsiella pneumoniae*, has led to the revival of polymyxin B use [2].

Current recommendations on the dosing of polymyxin B is largely based on convention, rather than robust pharmacokinetic evidence [1]. In accordance with the old literature, polymyxin B appears to be largely eliminated via the renal route [3]. However, recent pharmacokinetic studies have refuted this theory and have proven that nonrenal clearance predominates polymyxin B elimination [2, 4]. We described 2 patients with preexisting end stage renal failure, who had received nonattenuated doses of polymyxin B for the treatment of* Acinetobacter baumannii*-related infections.

## 2. Case Report 

The first patient, a 63-year-old Chinese man, 59 kg, had a background of hemodialysis-dependent end stage renal failure and had reported occasional voiding of urine 1-2 times per day as his baseline. He presented to the hospital with a 2-day history of fever, productive cough, shortness of breath, and chest pain. A chest radiograph revealed widespread infiltrates over the left lung and a septic workup showed elevated procalcitonin of 41.9 *μ*g/L and C-reactive protein (CRP) of 285 mg/L with normal total white cell count of 6.5 × 10^9^ cells/L. Upon admission, he was empirically initiated on intravenous (IV) cefepime for the treatment of healthcare-associated pneumonia in view of previous hospitalization less than 1 month ago. Unfortunately, the patient also developed a TIMI 3 ST-elevated myocardial infarction upon admission and hence underwent an emergency percutaneous coronary intervention. Postoperatively, he was hypotensive, requiring inotropic support, and was placed on sustained low efficiency dialysis (SLED).

He remained febrile despite one day of cefepime (*T*_max⁡_ of 38.4°C) with a further increase in CRP to 322 mg/L. Thus, antibiotics were escalated to IV meropenem and vancomycin. On day 4 of admission, although his fever had lysed, the total white cell count and procalcitonin level were observed to have increased to 16.28 × 10^9^   cells/L (95% neutrophils) and 47.1  *μ*g/L respectively. Blood cultures obtained on the day of admission yielded XDR* Acinetobacter baumannii*, sensitive to polymyxin B (MIC = 0.5 mcg/mL) only. Thus, meropenem and vancomycin were changed to polymyxin B.

Intravenous polymyxin B sulphate was given for 14 days at a dose of 750,000 units every morning and 500,000 units every evening (i.e., 1,250,000 units/day, approximately 21,000 units/kg/day) infused over 2 hours. Pneumonia was postulated to be the likely source of the* Acinetobacter baumannii *bacteremia. He was also initiated on concurrent nebulized colistin 2 MU every 8 hourly for 7 days. His preexisting vascular catheter was removed prior to polymyxin B initiation and cultured to rule out any possible catheter-related infections. The catheter tip was subsequently negative for bacterial growth.

Microbiological clearance of the blood cultures was documented on day 2 of polymyxin B treatment. The patient was weaned off the inotropes and was reverted to hemodialysis by day 3 of polymyxin B treatment. The patient remained afebrile and was hemodynamically stable. Improvements to his inflammatory markers were noted at the end of therapy; procalcitonin level and white cell count were reduced to 2.7 *μ*g/L and 12.19 × 10^9^ cells/L, respectively. Of note, there were no documented polymyxin B induced adverse events throughout the entire therapy. Signs of neurotoxicity were absent while the patient's residual renal function was preserved. He was still able to pass urine occasionally while being on diapers upon completion of the therapy.

The second patient is a 69-year-old, Vietnamese woman, 57 kg, with end stage renal failure on hemodialysis. She was transferred from a Vietnamese hospital for further management of MRSA line sepsis secondary to infected vascular catheter, which was already removed. Based on the limited information from the Vietnamese intensive care unit, she was mechanically ventilated and on inotropic support for at least 10 days prior to transfer. Upon arrival at our hospital, she was initiated on IV vancomycin and meropenem. She was also commenced on SLED in view of hypotension and renal failure.

Two endotracheal tube aspirates taken on separate days grew XDR* Acinetobacter baumannii *(with polymyxin B MIC of 0.5 mcg/mL). The chest radiograph showed extensive bilateral pulmonary consolidation. Her inflammatory markers were abnormal: procalcitonin of 3.2 *μ*g/L, CRP of 60.6 mg/L and total white cell count of 3.59 × 10^9^ cells/L.

Her antibiotics were switched to IV polymyxin B sulphate 750,000 units every 12 hourly (i.e., 1,500,000 units/day, approximately 26,000 units/kg/day) as a loading dose on the first day followed by 500,000 units every 12 hourly (i.e., 1,000,000 units/day, approximately 17,500 units/kg/day) on the second day. In addition, nebulized colistin 2MU every 8 hourly was also concurrently initiated for the treatment of ventilator-associated pneumonia (VAP).

Microbiological clearance was obtained from 2 negative sputum cultures taken on day 2 and day 5 of polymyxin B treatment. Efforts to wean the patient off the ventilator subsequently were futile. The chest radiograph on day 13 of polymyxin B treatment revealed worsening of bilateral consolidations and 2 sputum cultures on separate days grew* Stenotrophomonas maltophilia.*

The patient tolerated a total of 13 days of nonattenuated IV polymyxin B and 7 days of nebulized colistin. At the end of the therapy, her residual urinary output was preserved at an average volume of 70 mL per day.

## 3. Discussion 

To our knowledge, this is the first case report describing the use of nonattenuated doses of polymyxin B in patients with preexisting end stage renal failure.

In contrast to previously known pharmacokinetic data, 2 recent studies by Zavascki et al. and Sandri et al. on a group of critically ill patients demonstrated that the median urinary recovery of polymyxin B was <1% and 4.04% at steady state, respectively [2, 4]. In addition, Sandri et al. showed that the total body clearance of polymyxin B was not dependent on creatinine clearance, APACHE II score, or age [4]. Given diverse baseline demographics of the study patients, the total clearance of polymyxin B was best scaled by total body weight. Thus, dosage adjustment based on renal function was not advocated.

Attenuated doses of polymyxin B may potentially lead to treatment failure. A recent case report had described the implications of employing attenuated doses of polymyxin B in a critically ill and renally impaired patient for the treatment of intra-abdominal infection caused by XDR* Acinetobacter baumannii*. The patient died on day 4 of treatment and was noted to have received suboptimal doses of polymyxin B. The expected peak and trough concentrations were less than the susceptibility breakpoint of polymyxin B, hence, accounting for treatment failure [5].

From our case report, we have demonstrated the efficacy of using nonattenuated polymyxin B for the treatment of XDR* Acinetobacter baumannii *infections. Microbiological clearance was proven based on subsequent negative blood or sputum cultures. The first patient had also demonstrated significant clinical improvement while being on therapy.

Clinicians may be concerned with the high incidence of nephrotoxicity that was previously reported in the 1960s and 1970s. However, recent studies have showed that the incidence of nephrotoxicity is less common and severe presently compared to the old literature [6]. In a study conducted in Singapore, involving 26 patients who were administered with IV polymyxin B, none of the patients developed nephrotoxicity [7]. Additionally, another study from New York reported a nephrotoxicity rate of 14%, which was an overestimate in view of concurrent use of multiple nephrotoxic agents [8]. Another concern with polymyxins is neurotoxicity. It can be manifested as paresthesia, confusion, ataxia, and even neuromuscular blockade, leading to respiratory failure [9]. An old study reported an incidence of neurotoxicity in approximately 7% of noncystic fibrosis patients with the use of polymyxin E [10]. Mild neurological manifestations usually subside with prompt discontinuation of polymyxin [6].

From our observation, residual renal function of the 2 patients was present even after the completion of therapy with nonattenuated polymyxin B. With regard to the second patient with VAP, the inability to wean her off the ventilator was initially suspected to be secondary to the neurotoxicity of polymyxin B. However, it was ruled out subsequently as there were several factors that could have contributed to her dependence on ventilator which includes the new onset of* Stenotrophomonas maltophilia-*associated VAP and also critical illness polyneuropathy despite cessation of polymyxin B.

## 4. Conclusion 

It appears that the use of nonattenuated polymyxin B in hemodialysis-dependent patients may be efficacious and safe. Therefore, the dose of polymyxin B may not require dose adjustment in renally impaired patients. Further studies, such as randomized clinical trials will be required to support our observation.
